# Supplementary material for: Emission of coherent THz magnons in an antiferromagnetic insulator triggered by ultrafast spin–phonon interactions
Source: Nat Commun. 2023 Mar 31;14:1818. doi: 10.1038/s41467-023-37509-6 (PMC10066367; doi:10.1038/s41467-023-37509-6)
Supplement: Supplementary file 1 — Supplementary Information [file 41467_2023_37509_MOESM1_ESM.pdf]

Emission of coherent THz magnons in an antiferromagnetic insulator triggered by ultrafast spin-phonon interactions

E. Rongione[1,2], O. Gueckstock[3], M. Mattern[4], O. Gomonay[5], H. Meer[5], C. Schmitt[5], R. Ramos[6,7], T. Kikkawa[8], M. Mičica[2], E. Saitoh[6,8,9], J. Sinova[5], H. Jaffrès[1], J. Mangeney[2], S. T. B. Goennenwein[10], S. Geprägs[11], T. Kampfrath[3], M. Kläui[5,12,13], M. Bargheer[4,14], T. S. Seifert[3,*], S. Dhillon[2], R. Lebrun[1,*]

[1] Unité Mixte de Physique, CNRS, Thales, Université Paris-Saclay, F-91767 Palaiseau, France
[2] Laboratoire de Physique de l'Ecole Normale Supérieure, ENS, Université PSL, CNRS, Sorbonne Université, Université Paris Cité, F-75005 Paris, France
[3] Institute of Physics, Freie Universität Berlin, D-14195 Berlin, Germany
[4] Institut für Physik und Astronomie, Universität Potsdam, D-14476 Potsdam, Germany
[5] Institute of Physics, Johannes Gutenberg-University Mainz, D-55099 Mainz, Germany
[6] WPI-Advanced Institute for Materials Research, Tohoku University, Sendai J-980-8577, Japan
[7] Centro de Investigación en Química Biolóxica e Materiais Moleculares (CIQUS), Departamento de Química-Física, Universidade de Santiago de Compostela, Santiago de Compostela 15782, Spain
[8] Department of Applied Physics, The University of Tokyo, Tokyo J-113-8656, Japan
[9] Institute for AI and Beyond, The University of Tokyo, Tokyo J-113-8656, Japan
[10] Department of Physics, University of Konstanz, D-78457 Konstanz, Germany
[11] Walther-Meißner-Institut, Bayerische Akademie der Wissenschaften, D-85748 Garching, Germany
[12] Graduate School of Excellence Materials Science in Mainz (MAINZ), Staudingerweg 9, D-55128 Mainz, Germany
[13] Center for Quantum Spintronics, Department of Physics, Norwegian University of Science and Technology, N-7034 Trondheim, Norway
[14] Helmholtz-Zentrum Berlin für Materialien und Energie, Wilhelm-Conrad-Röntgen Campus, BESSY II, Albert-Einstein-Strasse 15, D-12489 Berlin, Germany

*Corresponding authors: tom.seifert@fu-berlin.de, romain.lebrun@cnrs-thales.fr

The Supplementary Material presents different THz signals for NiO(001)(10nm)/Pt(2nm) samples (S1), the Kerr imaging of NiO twin domains (S2), a pump power dependence on NiO/Pt THz emission (S3), the demonstration of the inverse spin Hall effect as the THz radiation emission process in NiO/Pt bilayers (S4), the magnetic field independent THz signal from NiO/Pt bilayers (S5), a detailed description of the modelling of the THz dynamics of the Néel vector, magneto-optical effects and discussions about potential spin-Seebeck contribution (S6), the dependence on linear polarization rotation (angle α) of NiO(111) films (S7), a detailed description of the ultrafast X-ray diffraction experiments and ultrafast strain modelling (S8), and an experimental temperature dependence of the NiO/Pt THz emission (S9).

S1. THz signals in different NiO(001)(10nm)/Pt(2nm) samples

We present in **Fig. S1** THz measurements for two different batch of NiO(001)(10nm)/Pt(2nm) samples. On the second sample (denoted as sample B, grown at 1.5 sccm of O_2), we could not measure any 1 THz oscillations as mapped in sample A (grown at 0.7 sccm of O_2). This evidence the crucial role of optimizing the NiO growth conditions, whilst the origin of this different behavior can arise from either a larger damping coefficient in NiO or a change of the magneto-strictive coefficient λ_{11}.

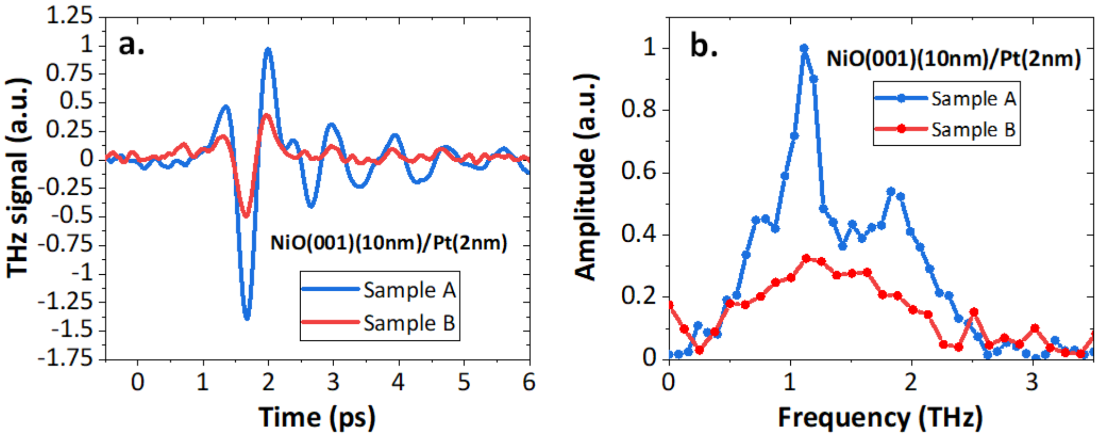

Fig. S1. (a) Time-domain THz signal from different samples of identical composition NiO(001)(10nm)/Pt(2nm). We respectively map 1 THz oscillations in sample A compared to sample B. (b) Fourier transform of the two time-domain THz-emission signals. The presence of 1 THz oscillations is marked only in sample A.

S2. Kerr imagery on NiO antiferromagnetic twin domains

To obtain domain arrangement and orientation on NiO thin films, we performed optical imaging of the antiferromagnetic *T*-domains using magneto-optical effects in an adapted Kerr microscope (following the approach described in Ref. [1]) as shown in **Fig. S2** for a NiO(001)(10nm)/Pt(2nm) sample. We identify three small as-grown antiferromagnetic *T*-domain (white contrast) oriented at 90° from the largest majority *T*-domain (grey contrast). The black spots are defects also imaged in the sample morphology. The thin film therefore presents large uniaxial domains that we estimate to be in average larger than 100 x 100 μm^2.

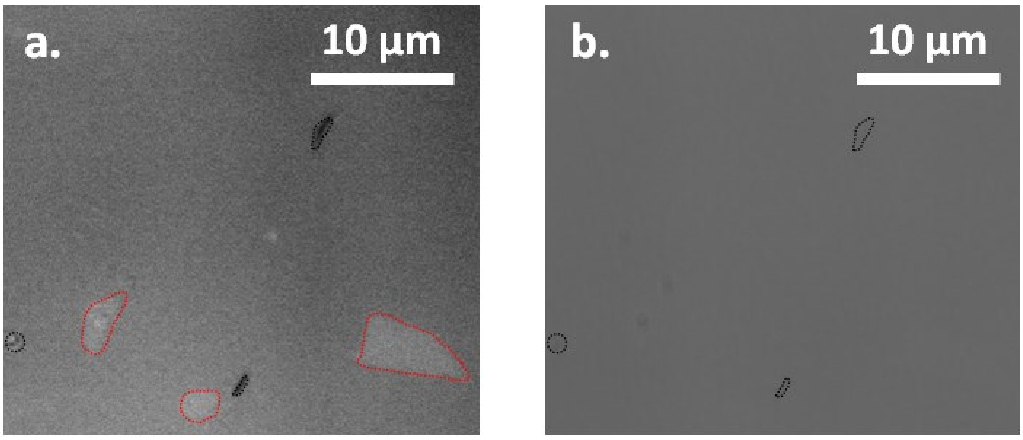

Fig. S2. Kerr birefringence imaging of the antiferromagnetic domains on NiO(001)(10nm)/Pt(2nm). (a) Birefringence difference imaging presenting two *T*-domains orientation with white contrast (circled in red) and grey (majority orientation, largest domain) and (b) sample morphology imaging indicating the defects at the surface of the sample (circled in black). The majority domain orientation (grey contrast) has an area estimated to be larger than 100 x 100 µm^2.

S3. Pump power dependence of the NiO/Pt THz emission

We present in **Fig. S3** the fluence dependence on the THz emission for NiO/Pt bilayers which shows linear trend as expected from spintronic based THz emission mediated by spin-charge conversion (SCC) emission mechanism [2]. In our study, we worked below the damage threshold.

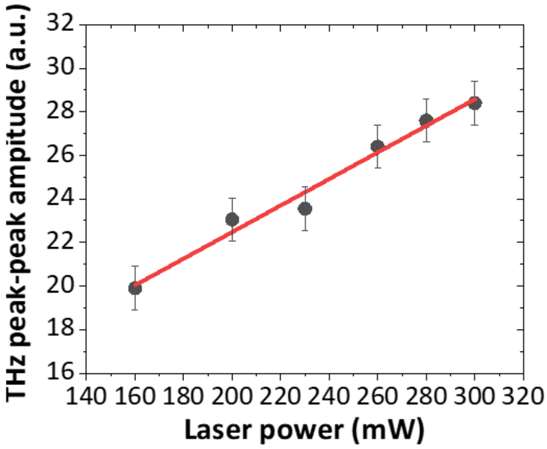

Fig. S3. Pump power dependence on the THz emission from NiO(111)(110nm)/Pt(10nm). The applied laser power ranges from 160 to 300 mW below the damage threshold. The beam diameter is around 200 µm.

S4. THz emission mechanism originating from inverse spin Hall effect

To clearly identify the inverse spin Hall effect (ISHE) as the main mechanism allowing the THz emission from generated magnon flow from NiO, we performed two experimental checks. First, on (001) oriented thin films, **Fig. S4a** presents the THz emission from NiO/Pt, and NiO/W and NiO/Ta bilayers on a same (001) thin film. The presence of a phase reversal of the generated

THz signal for Pt (**Fig. S4a-b**), and W (**Fig. S4a)** and Ta (**Fig. S4b)** based bilayers, indicates that the spin-charge conversion (SCC) occurring via ISHE is responsible for the detected THz emission according to $j_c \propto \theta_{\mathrm{SHE}}(j_s \times m)$ and given the opposite spin-Hall angle between Pt, and W and Ta. The smaller amplitude obtained in NiO/W and NiO/Ta arises potentially from i) a different spin-mixing conductance and/or ii) a smaller value of the spin Hall angle. One can nevertheless notice here that the presence of the oscillations also in the W case despite the lower signal amplitude.

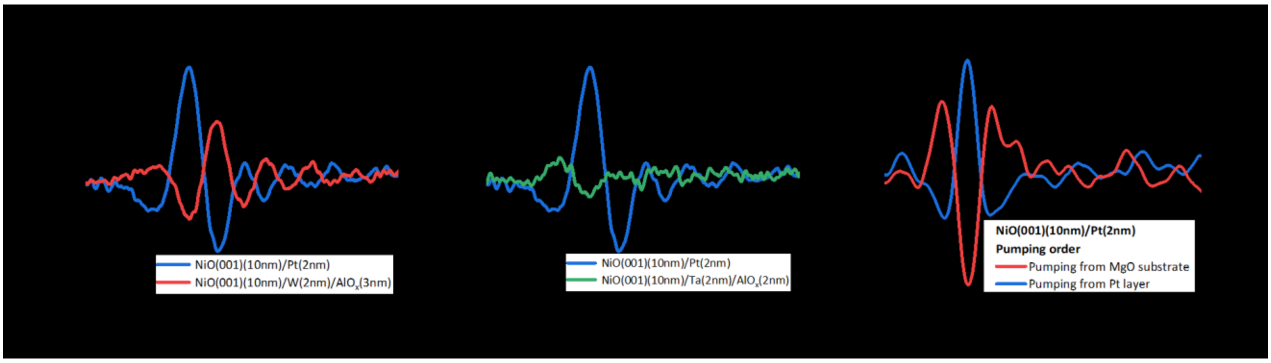

Fig. S4. Identification of ISHE as the emission mechanism in (001) thin films. (a-b) Role of the spin Hall angle in (001) oriented NiO. Comparison between (a) NiO/Pt vs NiO/W and (b) NiO/Pt vs NiO/Ta. (c) Emitted THz signal when reversing the sample surface facing the optical pump (*i.e.* pumping first from Pt or from the MgO substrate side). The phase reversal is in line with spin-charge conversion in Pt. In the case of pumping from the MgO substrate side, the signal has been shifted in time for clarity.

Secondly, we also collected the THz emission with different pumping direction to discriminate between electric or magnetic dipolar emission. **Fig. S4c** presents the THz emission when pumping from either the front (Pt) or substrate (MgO) side. It appears the generated THz polarization is reversed, which is also in line with a THz emission emerging from SCC in the Pt layer and not with a potential dipolar emission from the dynamics of the AFM moments. One must notice the crucial role of the spin-transparency of the NiO/Pt interface in this SCC process, with a measured spin-mixing conductance of about 10^{14} $\Omega^{-1}.m^{-1}$ (Ref. [3]) as large as for ferromagnetic materials.

Lastly, we have replaced, for a (111) oriented NiO sample, the capping layer Pt(10nm) by W(10nm), which has a negative spin Hall angle $\theta_{\mathrm{SHE}}^{\mathrm{W}}$ about the same amplitude as the positive spin Hall angle of Pt $\theta_{\mathrm{SHE}}^{\mathrm{Pt}}$. We also observed a phase reversal (see **Fig. S5**), in line with spin-to-charge conversion processes.

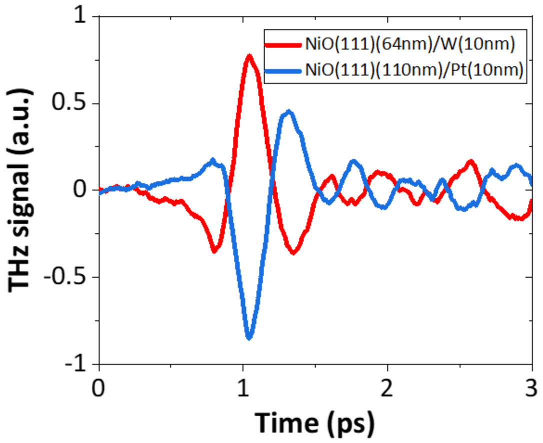

Fig. S5. Identification of ISHE as the emission mechanism. a) THz emission from NiO(111)(110nm)/Pt(10nm)/AlO$_x$(1.5nm) (blue) and NiO(111)(64nm)/W(10nm)/AlO$_x$(1.5nm) (red) present a phase reversal which agrees with identifying spin Hall effect as the emission mechanism in these bilayers

S5. Magnetic field dependence of the THz emission

We performed the magnetic field dependence measurement of the THz emission by applying a static magnetic field of about 200 mT in the sample plane (along $\boldsymbol{e}_x$ and $\boldsymbol{e}_y$ direction) as shown in **Fig. S6**. We measured an identical THz trace when applying (or not) an external magnetic field. The THz-emission signal shows a time-domain 1 ps oscillations in all cases. This independence of THz emission with respect to applied magnetic field is in line with antiferromagnetic origin of the THz emission. It allows us to discard the presence of an uncompensated interface, which could have resulted in spin current injection via ultrafast demagnetization. One must also notice that only X-ray magnetic linear dichroism (XMLD) (and not X-ray magnetic circular dichroism - XMCD -) signals were measured on these samples [4].

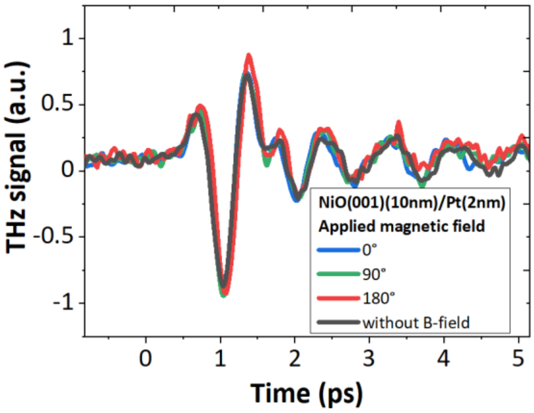

Fig. S6. Magnetic field dependence on NiO(001)(10nm)/Pt(2nm). Applied in-plane magnetic field is around 200 mT.

S6. Modelling of the NiO spin dynamics under various torque excitations

In this section, we introduce NiO antiferromagnetic ordering before considering the dynamics of the Néel vector. We then introduce magneto-optical torque and thermo-magneto-elastic effect and their respective symmetries which allow THz emission.

NiO material description.

NiO is an easy-plane antiferromagnet with the Néel temperature of 523 K (Ref. [5]). Below the Néel temperature, the magnetic spins lie in (111) planes where $\mathbf{M}_1$ and $\mathbf{M}_2$ describe opposite spin ordering between adjacent planes in equilibrium state. Magnetic structure can be realized in form of four possible *T* domains that are distinguished by orientation of easy magnetic planes [6]. Orientation of the magnetic moments in easy magnetic plane and *T*-domain structure depend on the film orientation. In NiO(111) samples, the growth conditions favor formation of a single *T* domain with $\mathbf{M}_1 \uparrow\downarrow \mathbf{M}_2$ aligned along one of the three equivalent $[11\bar{2}]$ directions in (111) plane, thus forming three equivalent *S* domains. NiO(001) films show multidomain structure with all four possible *T* domains. However, pronounced out-of-plane deformation [4] removes *S* domains and stabilizes the single equilibrium orientation along $[5\,5\,\bar{1}9]$ within each of *T* domain. We thus consider this initial AFM state in our theoretical modelling.

Equation of motion of the antiferromagnetic order

Under excitation, we described the antiferromagnetic order dynamics by:

$$\boldsymbol{n} \times \left(\ddot{\boldsymbol{n}} + 2\gamma_{\mathrm{AF}}\dot{\boldsymbol{n}} - c^2\Delta\boldsymbol{n} + \omega_{\mathrm{AF}}^2(T)\,\boldsymbol{\delta n}\right) = \gamma^2 H_{\mathrm{ex}}\,(\boldsymbol{n} \times \boldsymbol{\Gamma}), \qquad \text{(S1)}$$

where $\omega_{\mathrm{AF}}^2 = \gamma^2 H_{\mathrm{ex}} H_{\mathrm{an}}$ is the angular frequency of the magnetic oscillations depending on the temperature T with γ the gyromagnetic ratio, H_{ex} the exchange field that keeps the magnetic sublattice moments antiparallel, $M_s \boldsymbol{H}_{\mathrm{an}}(\boldsymbol{n}) = -\partial w_{\mathrm{an}}/\partial \boldsymbol{n}$ the magnetic anisotropy field, w_{an} is the density of the magnetic energy, $M_s/2$ is the sublattice magnetization, γ_{AF} is the damping constant and c is the limiting magnon velocity. **Eq. (1)** of the main text is defined by linearization of **Eq. (S1)** with respect to small deviations $\boldsymbol{\delta n}$ of the Néel vector from equilibrium orientation $\boldsymbol{n_0}$. We picture in **Fig. S7** the projection of the initial Néel vector $\boldsymbol{n_0}$ and small deviations $\boldsymbol{\delta n}$ into the $(1\bar{1}0)$ and (001) plane.

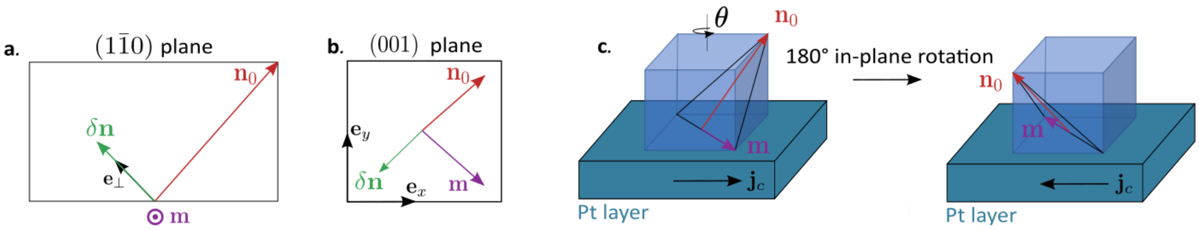

Fig. S7. Projection into the (a) $(1\bar{1}0)$ plane and (b) (001) plane of the equilibrium orientation of the Néel vector $\boldsymbol{n_0}$, the Néel vector variations $\boldsymbol{\delta n}$ and of dynamics magnetization $\boldsymbol{m}$ generated when exciting the high frequency THz mode. These two components ($\boldsymbol{\delta n}, \boldsymbol{m}$) oscillate with time along the represented directions. (c) Under rotation by 180°, the projected magnetization $\boldsymbol{m}$ is reversed leading to an opposite generated charge current j_c.

We associate the spin current j_s injected in the Pt with the spin accumulation produced by magnetization $\boldsymbol{m}$ at the NiO/Pt interface. The polarization of the emitted THz signal $\boldsymbol{E}_{\mathrm{out}}$ is then parallel to the current density *i.e.* we have $\boldsymbol{E}_{out} \propto \boldsymbol{j}_c \propto 2\theta_{\mathrm{SH}} e \cdot j_s \boldsymbol{e}_z \times (\boldsymbol{n_0} \times \boldsymbol{e}_\perp)/\hbar$ in the Pt layer [7], where θ_{SH} is the spin Hall angle, e is the electron charge, $\hbar$ is the reduced Planck constant. We further display in this part the two contributions allowing the excitation of THz dynamics depending on the two studied orientations (001) or (111). Either the excitation mechanism needs to have the appropriate symmetry with respect to the orientation to excite

the Néel mode or the generated spin current j_s would need to have the correct symmetry (*i.e.* injection of angular momentum along the interface normal) to be efficiently converted via ISHE.

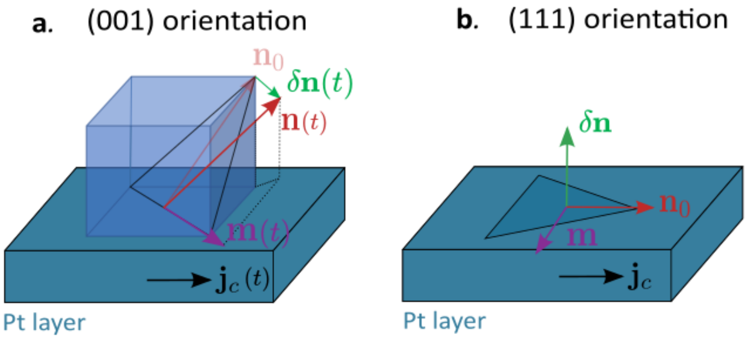

Fig. S8. Out-of-plane mode (of frequency around 1 THz) excitation geometries for (001) orientation (a) and (111) orientation (b). The Néel vector $\boldsymbol{n}(t) = \boldsymbol{n_0} + \boldsymbol{\delta n}(t)$ is represented in red, the variations of the Néel vector $\boldsymbol{\delta n}(t)$ are represented in green and the magnetization is projected in purple**.** The antiferromagnetic plane including Néel vector is represented as a triangle.

We display in **Fig. S8** the out-of-plane mode excitation configurations leading to the generation of charge current in Pt from NiO (001) and (111). In order to induce the THz mode, the torque $\boldsymbol{\Gamma}$ should have a component parallel to $\boldsymbol{e}_\perp$ (otherwise, the low frequency mode is excited) while in order to excite a charge current in the Pt layer, the magnetization $\boldsymbol{m}$ should have an in-plane component, which is achieved only for the 1 THz mode in the case of (111) oriented films.

We describe theoretically below in more detail the three different torques discussed in the main text, *i.e.* an off-resonant optical spin torque with the symmetry of the inverse Cotton-Mouton effect (ICME) and thermo-magneto-elastic effects which can generate a dynamical net moment $\boldsymbol{m}$ leading to the generation of a non-zero THz signal by the inverse spin-Hall effect:

Off-resonant optical spin torque via Inverse Cotton-Mouton effect (ICME). The interaction between light and matter is described phenomenologically by the Hamiltonian

$$H_{\text{int}} = g_{ijkl} n_i n_j E_k E_l^*. \tag{S2}$$

The structure of the fourth rank tensor of phenomenological coefficients g_{ijkl} is defined by the symmetry of the paramagnetic state of the crystal. In the frame related with crystallographic axes $\boldsymbol{e}_x$ || $[100]$, $\boldsymbol{e}_y$ || $[010]$, $\boldsymbol{e}_z$ || $[001]$, the non-trivial components are $g_{11} = g_{22} = g_{33}$, $g_{44} = g_{55} = g_{66}$, and $g_{12} = g_{23} = g_{31}$ (Voigt notation). The torque between optical pump and antiferromagnetic order is defined as $\boldsymbol{\Gamma} = \partial H_{\text{int}} / \partial \boldsymbol{n}$, so that:

$$\Gamma_i = g_{ijkl} n_{0j} E_k E_l. \tag{S3}$$

The out-of-plane mode (1 THz) can be excited either in NiO(111) and in NiO(001) samples. For NiO(111), the component of the torque $\boldsymbol{\Gamma}$ in $\boldsymbol{e}_\perp$ direction is:

$$\boldsymbol{\Gamma} \cdot \boldsymbol{e}_\perp = \frac{E_{\text{in}}^2}{3\sqrt{2}} [g_{44} + (3g_{44} - 2g_{11} + 4g_{12})\cos 2\alpha], \tag{S4}$$

where we assume that the light is linearly polarized and α is the angle with respect to equilibrium orientation of the Néel vector. This result is consistent with that of Ref. [8]. For NiO(001), we have:

$$\boldsymbol{\Gamma}\cdot\boldsymbol{e}_{\perp}=\frac{E_{\text{in}}^{2}}{3\sqrt{2}}[2(g_{11}-2g_{12})+4g_{44}\sin2\alpha]. \tag{S5}$$

By introducing the notations $g_1 \equiv 2(g_{11}-2g_{12})$, $g_2 \equiv g_{44}$ and $P_{\text{in}}=E_{\text{in}}^{2}c_{\text{light}}$, we get:

$$\boldsymbol{\Gamma}\cdot\boldsymbol{e}_{\perp}=P_{\text{in}}[g_2+(3g_2-g_1)\cos2\alpha]/(3\sqrt{2}c_{\text{light}}) \text{ for NiO(111)},$$

$$\boldsymbol{\Gamma}\cdot\boldsymbol{e}_{\perp}=P_{\text{in}}[g_1+4g_2\sin2\alpha]/(3\sqrt{2}c_{\text{light}}) \text{ for NiO(001)}.$$

Furthermore, we then expect the amplitude of the emitted THz signal $\boldsymbol{E}_{\text{out}}$ to scale with the NiO thickness (up to the magnon spin-diffusion length of around 100 nm in NiO [9]) and with the incoming laser power as P_{in} which are both in line with the observations of **Fig. 3**. Though ICME is non-zero for both NiO(001) and NiO(111), its value and angular dependence is defined by different combinations of the coefficients g_{ijkl} Hence, we expect a different ICME contribution for NiO(001). We recall that inverse Faraday contribution can be excluded from the pump polarization dependence. Our experimental results are, in this regard, in line with Refs. [8,10], and demonstrate the stronger off-resonant optical spin torque contributions from (111) samples.

Thermo-magneto-elastic effect. Magneto-elastic coupling between the components of the Néel vector and strain tensor ε_{kl} is described by the Hamiltonian

$$H_{\text{int}}=\lambda_{ijkl}n_in_j\varepsilon_{kl} \tag{S6}$$

where the fourth rank tensor λ_{ijkl} of magneto-elastic constants has the same structure as tensor g_{ijkl}. Inhomogeneous laser-induced heating creates an out-of-plane strain component $\varepsilon_{zz}(z,t)$ whose time dependence $\varepsilon_{zz}^{\text{NiO}}(t)$ (integrated over the NiO thickness) is shown in **Fig. 4b** of the main text. The corresponding local component of the torque $\boldsymbol{\Gamma}$ in $\boldsymbol{e}_{\perp}$ direction is:

$$\boldsymbol{\Gamma}\cdot\boldsymbol{e}_{\perp}=\lambda_{11}n_{0z}\varepsilon_{zz}(z,t)e_{\perp z}. \tag{S7}$$

In NiO, we have $\lambda_{11}=3\times10^{7}$ J.m^{-3} according to Schmitt *et. al.* [4]. In case of (111) NiO samples, $n_{0z}=0$ and thermo-magneto-elastic torque vanishes as shown in **Fig. S9**. For (001) films $\boldsymbol{n}_{0z}\cdot\boldsymbol{e}_{\perp z}=0.46$, and the thermo-magneto-elastic torque produces a pronounced effect. From the amplitude of the maximum out-of-plane strain wavefront $\varepsilon_{zz}=6\times10^{-6}$, we estimated the tilt of the Néel vector to be around $\phi=\arccos(\boldsymbol{n}(t)\cdot\boldsymbol{e}_z)=\varepsilon_{zz}^{\text{NiO}}\lambda_{11}\gamma/(2\gamma_{\text{AF}})\simeq0.3°$ for the THz-TDS measurements. In this case, the magnetization should be of the order of 1 A/m following the data from Ref. [4].

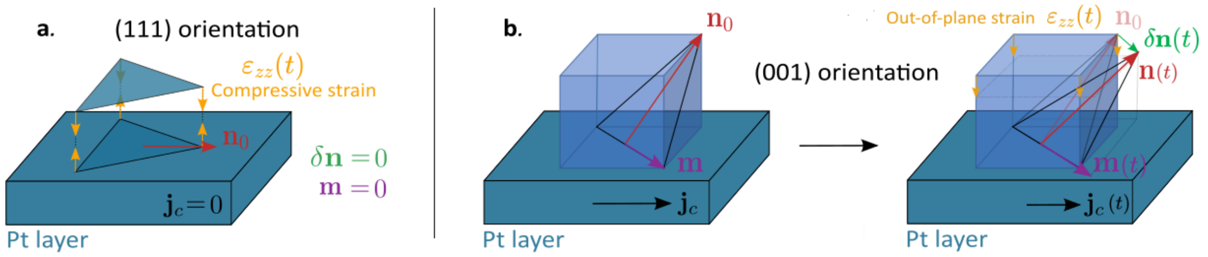

Fig. S9. Symmetry of the thermo-magneto-elastic induced torque. (a) In case of (111) orientation, the strain wave cannot induce an excitation of the Néel vector as $\boldsymbol{\Gamma} \cdot \boldsymbol{e}_z = 0$. (b) For (001), the Néel vector undergo magneto-elastic reduction $\boldsymbol{n}(t) = \boldsymbol{n}_0 + \boldsymbol{\delta n}(t)$ due to a non-vanishing torque under an applied out-of-plane strain wave $\varepsilon_{zz}(t)$.

Its spin signal then depends on the orientation of the Néel vector with respect to crystallographic axes. The sign of the out-coming signal, however, changes sign for the reversed film orientation (as shown in **Fig. 2b** and pictured in **Fig. S7**), leading to the opposite direction of the spin current into Pt layer (in line with **SM4**). On the contrary, the emitted signal is proportional to the intensity of pump pulse and does not depend on orientation of light polarization. The amplitude of the effect is inversely proportional to the thickness of NiO layer, as excitation of magnons takes place at the NiO/Pt interface. The timescale is defined by the slowest process, thermal heating of Pt and falls into picosecond range in line with the observations of **Fig. 4b**.

Spin-Seebeck effect contributions (SSE). First, it should be noted that the standard bulk spin-Seebeck effect, present in magnetic systems for which a magnon flux carries angular momentum (ferromagnets, easy-axis antiferromagnets under applied field), seems to be irrelevant in the NiO/Pt system. A generated thermal gradient in the NiO (hot interface with Pt and cold interface with MgO) is a necessary factor but not the only element that needs to be considered for the spin-Seebeck effect. In NiO, although it possesses a sizeable macroscopic AFM ordering [11], the two magnon branches are non-degenerate at zero magnetic field [12,13], which excludes the spin-Seebeck excitation mechanism for building a net spin current. Moreover, the weak temperature dependence of the NiO(001)(10nm)/Pt(2nm) THz emission (see **SM9**) does not fit the theoretical expectations for SSE [13].

It should then be noted that the symmetry of (001) films allows for a second spin-Seebeck contribution proportional to the temperature gradient at the NiO/Pt interface and to spin-spin correlations, with the same symmetry as the thermo-magneto-elastic effect. In order to describe this contribution, we follow the approach of Ref. [14] used for YIG/Pt systems and consider a torque induced by spin fluctuations $\boldsymbol{s}_{\mathrm{Pt}}$ in the Pt layer:

$$\boldsymbol{\Gamma} = H_{\mathrm{curr}}\,(\boldsymbol{s}_{\mathrm{Pt}} \times \boldsymbol{n}), \tag{S8}$$

where H_{curr} is a phenomenological constant whose value depends on the properties of the NiO/Pt interface. The average of the spin fluctuations is $\langle \boldsymbol{s}_{\mathrm{Pt}}(t) \rangle = 0$, however, they produce non-zero fluctuations of the Néel vector. In the frequency domain, we have:

$$\boldsymbol{\delta n}(\omega) = \frac{\gamma^2 H_{\mathrm{ex}} H_{\mathrm{curr}} \boldsymbol{s}_{\mathrm{Pt}}(\omega)}{\omega^2 + 2i\gamma_{\mathrm{AF}}\omega - \omega_{\mathrm{AF}}^2}. \tag{S9}$$

By substituting **Eq. (S9)** into **Eq. (S8)** and accounting for a second order term in $\boldsymbol{\delta n}$ we get an expression of the torque

$$\boldsymbol{\Gamma}(\omega) = \frac{\gamma^2 H_{\mathrm{ex}} H_{\mathrm{curr}}^2}{\omega^2 + 2i\gamma_{\mathrm{AF}}\omega - \omega_{\mathrm{AF}}^2} \langle \boldsymbol{s}_{\mathrm{Pt}}(\omega) \times \boldsymbol{n_0}(\boldsymbol{s}_{\mathrm{Pt}}(\omega) \cdot \boldsymbol{n_0}) \rangle. \quad \text{(S10)}$$

This torque is associated with the spin current $\mathbf{j}_{s1} \propto \langle \boldsymbol{s}_{\mathrm{Pt}}(t) \times \boldsymbol{n_0}(\boldsymbol{n_0} \cdot \boldsymbol{s}_{\mathrm{Pt}}(t')) \rangle$ flowing from Pt to NiO layer. According to the fluctuation-dissipation theorem, the time-dependence of spin-spin correlations $\langle \boldsymbol{s}_{\mathrm{Pt}}(t) \boldsymbol{s}_{\mathrm{Pt}}(t') \rangle \propto T_{\mathrm{Pt}}(t)\delta(t - t')$ is defined by the temperature of the Pt layer.

In a similar way, thermal fluctuations of the magnetization, $\boldsymbol{m} = \boldsymbol{n_0} \times \dot{\boldsymbol{\delta n}}/(\gamma H_{ex})$, in NiO exert a torque on Pt spins and create spin current $\mathbf{j}_{s2}$ in the opposite direction. The value of the spin current is proportional to the temperature T_{NiO} of NiO layer. In equilibrium ($T_{\mathrm{Pt}} = T_{\mathrm{NiO}}$), both spin currents are equal and compensate each other, $\mathbf{j}_{s1} = -\mathbf{j}_{s2}$. In presence of a small temperature gradient, the resulting spin current, $\mathbf{j}_{s1}+\mathbf{j}_{s2} \approx \mathbf{j}_{s1}(T_{\mathrm{Pt}} - T_{\mathrm{NiO}})/T$, is nonzero and creates a torque $\boldsymbol{\Gamma} \propto \langle \boldsymbol{s}_{\mathrm{Pt}}(t) \times \boldsymbol{n_0}(\boldsymbol{n_0} \cdot \boldsymbol{s}_{\mathrm{Pt}}(t')) \rangle \, \nabla T(t)$ on NiO spins proportional to the temperature gradient at the NiO/Pt interface. Although this non-linear (interfacial) spin-Seebeck effect has the same symmetry as the thermo-magneto-elastic effect, it shows a different time-dependence of the generated THz signal.

Numerical simulations. We then model the magnetization dynamics of the NiO triggered by the torque contributions associated with non-linear (interfacial) SSE and thermo-magneto-elastic effect in the (001) films using the Eq. (S1). To model the THz response, we use as input parameters the temperature and strain profile extracted by fitting the URSM data using the strain model presented in **SM7**. We used the strain profile $\varepsilon_{zz}(z,t)$ to calculate the thermo-magneto-elastic torque (S10) in NiO layer and plug it into dynamic Eq. (S4) to calculate the corresponding magnetization $\boldsymbol{m} = \boldsymbol{n_0} \times \dot{\boldsymbol{\delta n}}/(\gamma H_{ex})$ of the several near-interface NiO layers that contribute into the optical response E_{MAS}. We separately calculated the non-linear (interfacial) spin-Seebeck effect using the temperature profile extracted from the modelling in **SM7**.

We then present in **Fig. S10** the numerical simulations presenting the THz response of both component contributing to the overall modelling shown in **Fig. 1b**. It reads $E_{\mathrm{THz}} = a\, E_{\mathrm{MAS}} + b\, E_{\mathrm{T}}$ where E_{MAS} and E_{T} represent respectively the magneto-acoustic strain (Fig. **S10a**) and the temperature contributions according to the non-linear (interfacial) SSE (Fig. **S10b**) estimated in the presented fitting for a=-0.3 and b=0.7. It is to be noted that both components present a THz response centred around the out-of-plane magnon mode. The presence of oscillations at long timescale is assured by the magneto-acoustic strain contribution while the broadband contribution is fitted mainly via the temperature contribution. One must notice that the timescales of the modelled magnetization response are in line with the experimental THz response (**Fig. 4b**) and with the modelled ultrafast strain and temperature responses (**Fig. 4b** and **SM8**).

To simulate magnetic dynamics in NiO layer, we solved **Eq. (1)** of the main text by assuming that $\omega_{\mathrm{AF}}/2\pi$ =1.08 THz, $\gamma_{\mathrm{AF}}/2\pi$ = 0.16 THz (corresponding to a Gilbert damping of $\alpha = 0.006$), magnon velocity c = 10 nm/ps.

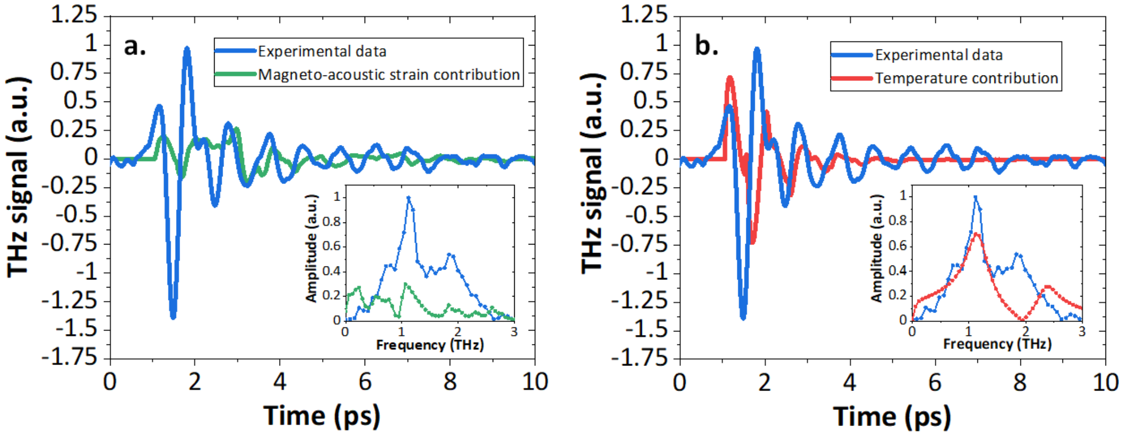

Fig. S10. Magnonic THz contribution fitted according to the magnetic dynamics in a NiO(001)(10nm)/Pt(2nm) layer. (a) Magneto-acoustic strain contribution and (b) temperature contribution. Insets present the respective contributions in the frequency domain.

S7. Linear pump polarization dependence on (111) films

Complementary to (001) oriented NiO films linear pump polarization dependence, we performed the same polarization dependence on NiO(111)(110nm)/Pt(2nm) sample as illustrated in **Fig. S11**. Contrary to the polarization independent THz emission measured for (001) films, we measured a cos(2α) dependence on the linear pump polarization, in line with magneto-optic excitation as discussed in the main manuscript and in Refs. [10,15]. Here, $\alpha = 0°$ corresponds to a p-polarized pump while we detect p-polarized THz component. The sample is placed so that $[1\bar{2}1]$ is along the s-direction. In this view, the reduced THz amplitude found for the circular pump polarization arises from the fact that pump electric field can be written as $\boldsymbol{E^{circ}_{pump}} = \frac{\boldsymbol{1}}{\sqrt{2}}(\boldsymbol{E^{p}_{lin}} + \boldsymbol{iE^{s}_{lin}})$. Thus, as we only map the p-polarized THz component, we measure a THz signal which is half the amplitude recovered for a fully p-polarized pump.

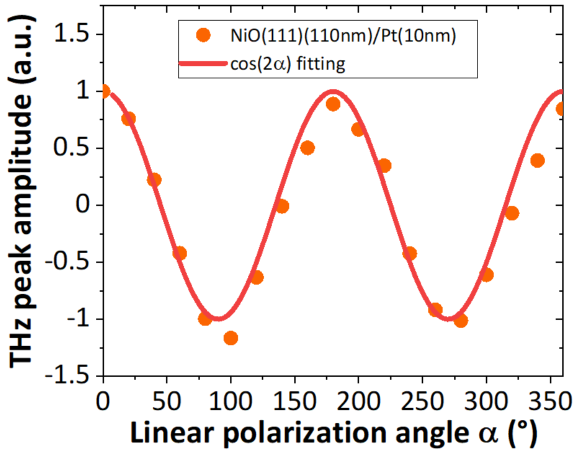

Fig. S11. Linear polarization dependence on NiO(111)(110nm)/Pt(10nm). The linear laser polarization is rotated by an angle α with respect to initial p-polarization.

S8. Ultrafast Reciprocal Space Mapping (URSM) experiments and modelling transient strain

Determination of transient strain in NiO

The time-resolved out-of-plane strain of the NiO layer is given by the relative change of the Bragg peak position in reciprocal space with the time delay t:

$$\varepsilon_{zz}^{\mathrm{NiO}}(t) = \frac{q_z(t < 0\ \mathrm{ps}) - q_z(t)}{q_z(t < 0\ \mathrm{ps})} \tag{S11}$$

Fig. S12 shows the reciprocal space around the (004) reflection of NiO as measured during the time-resolved experiment. The projection on the respective directions of the reciprocal space q_z and q_x (gray circles) consist of a background from the MgO substrate and the NiO Bragg peak. The determination of the position of the NiO Bragg peak along q_z and the corresponding expansion require the substration of the MgO background. We model the background from the diffraction of the substrate truncation rod by a Lorentzian fitted the intensity below 5.93 Å and above 5.98 Å. The difference between the total intensity and the modelled substrate background (black dashed line) determines the NiO Bragg peak (blue circles). Its time-dependent position along q_z (vertical gray dashed line) is determined by a center-of-mass analysis of the reciprocal-space maps recorded at each pump-probe delay.

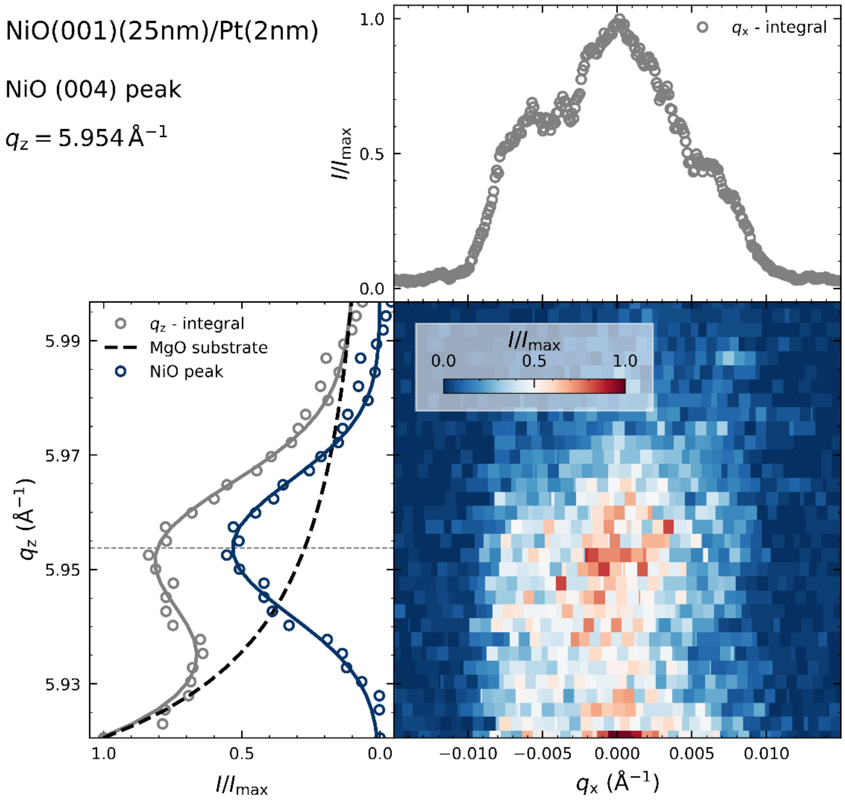

Fig. S12. Reciprocal space map (RSM) around the (004) reflection of a 25 nm NiO film on MgO covered with Pt(2nm). The rising intensity at the small angle side of the RSM originates from the MgO substrate Bragg peak, which is cut from the figure due to its 100 times higher intensity. The left panel displays the extraction of the NiO Bragg peak (blue circles) by integrating the intensity along the q_x-direction in reciprocal space (grey circles) and subtracting the modelled background provided by the MgO substrate (black dashed line). The blue and grey solid line describe the fitted NiO Bragg peak and its superposition with the background, respectively. The vertical dashed grey line denotes the determined position of the NiO Bragg peak along q_z which determines the out-of-plane strain of the NiO layer $\varepsilon_{zz}^{\mathrm{NiO}}$.

Transient strain modelling

We use the modular Python toolbox *udkm1Dsim* [16] to model the measured mean strain of the NiO layer, which provides access to the spatio-temporal temperature and strain used for the simulation of the THz signal. The absorbed energy in the Pt capping layer drives a transient out-of-plane strain $\varepsilon_{zz}(z,t) = \frac{\partial u}{\partial z}(z,t)$ within the sample structure according to the one-dimensional (1D) wave equation of the displacement field $u(z,t)$ with a source term given by the laser-induced stress $\sigma_{ext}(z,t)$:

$$\frac{\partial^2 u}{\partial t^2} - v_s^2 \frac{\partial^2 u}{\partial z^2} = -\frac{\partial \sigma_{\mathrm{ext}}}{\partial z} \tag{S12}$$

where v_s refers to the sound velocity. In general, the laser-induced stress is given by the product of the energy density $\rho^Q(z,t) = C \cdot \Delta T$ and the material-specific Grüneisen constant Γ for each subsystem, *i.e.* electrons and phonons [17]. We use the literature values of the thermo-elastic constants for the considered materials in **Table S1**. Since the electronic subsystem is only relevant in the Pt capping layer, we simplify the model by only considering one spatio-temporal temperature rise denoted as $\Delta T(z,t)$. We model the laser-induced temperature rise (see **Fig. S13a**) by first calculating the deposited energy in Pt and the subsequent transport via 1D heat diffusion. To account for a finite stress rise time in Pt due to electron-phonon coupling, we model the stress in Pt driving the strain wave by [18]:

$$\sigma_{\mathrm{ext}}^{\mathrm{Pt}}(z,t) = \rho_{\mathrm{Pt}}^{Q}(z,t) \cdot \Gamma_{\mathrm{ph}} \left(1 - \frac{\Gamma_{\mathrm{el}}}{\Gamma_{\mathrm{ph}}} e^{-\frac{t}{\tau_{\mathrm{el-ph}}}}\right) \tag{S13}$$

Inserting the resulting spatio-temporal laser-induced stress into **Eq. (S12)** results in the spatio-temporal strain displayed in **Fig. S13b**. We use dynamical X-ray diffraction theory to calculate a NiO Bragg peak from the modelled spatio-temporal strain in NiO. The results of this modelling correspond to the modelled transient mean strain of NiO represented as a blue line in **Fig. 4b**, which is in line with the experimental data points obtained by UXRD (from the time-dependent shift of the Bragg peak in reciprocal space).

	**Pt**	**NiO**	**MgO**
C $(\mathrm{J.cm^{-3}.K^{-1}})$	2.85 (Ref. [19])	4.2 (Ref. [20])	3.32 (Ref. [21])
κ $(\mathrm{W.m^{-1}.K^{-1}})$	71 (Ref. [22])	31 (Ref. [20])	50 (Ref. [23])
ρ $(\mathrm{g.cm^{-3}})$	21.45	6.79	3.58
v_s $(\mathrm{nm.ps^{-1}})$	4.2 (Refs. [24,25])	6.35 (Ref. [26]) (6.99)	9.12 (Ref. [27])
Γ_{el}	1.6 (Ref. [28])	-	-
Γ_{ph}	2.6 (Ref. [28])	0.9 (Refs. [29,30])	1.6 (Ref. [31])
$\tau_{\mathrm{el-ph}}$ (fs)	500 (Ref. [32])	-	-

Table S1. Thermoelastic constants table for Pt, NiO and MgO. C is the phonon heat capacity, κ is the heat conductivity, ρ is the mass density, v_s is the sound velocity (for NiO, we used a different sound velocity value given in brackets), Γ_{el} and Γ_{ph} are respectively the electronic (phononic) Grüneisen constant and $\tau_{\mathrm{el-ph}}$ is the electron-phonon coupling time.

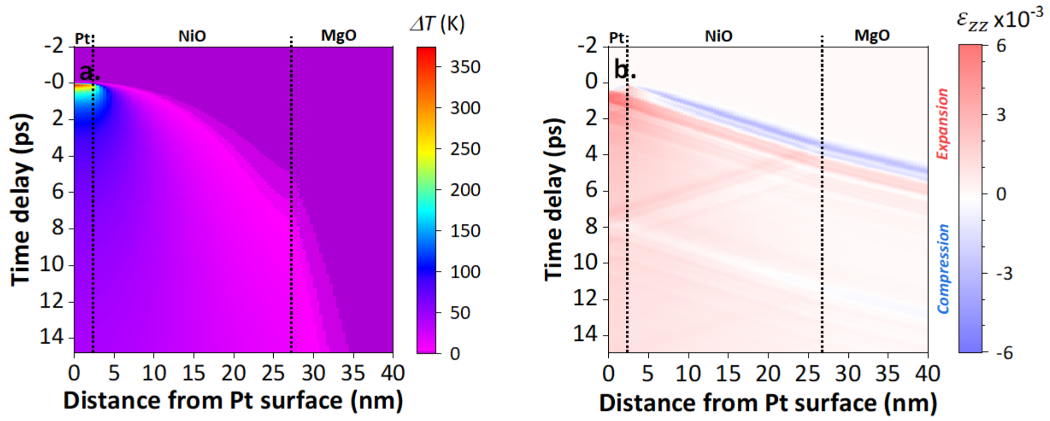

Fig. S13. Modelled spatio-temporal (a) temperature change $\Delta T(t,z)$ and (b) out-of-plane strain $\varepsilon_{zz}(t,z)$ for MgO/NiO(001)(25nm)/Pt(2nm). The vertical dashed lines denote the interfaces between the different layers. We refer t as the delay time and z the distance from the top Pt surface.

S9. Temperature dependence below and above Néel order on the THz emission

We performed in **Fig. S14** temperature dependence on the NiO(001)(10nm)/Pt(2nm) THz emission. The obtained dependence is not in in line with spin Seebeck effect as described in Ref. [13].

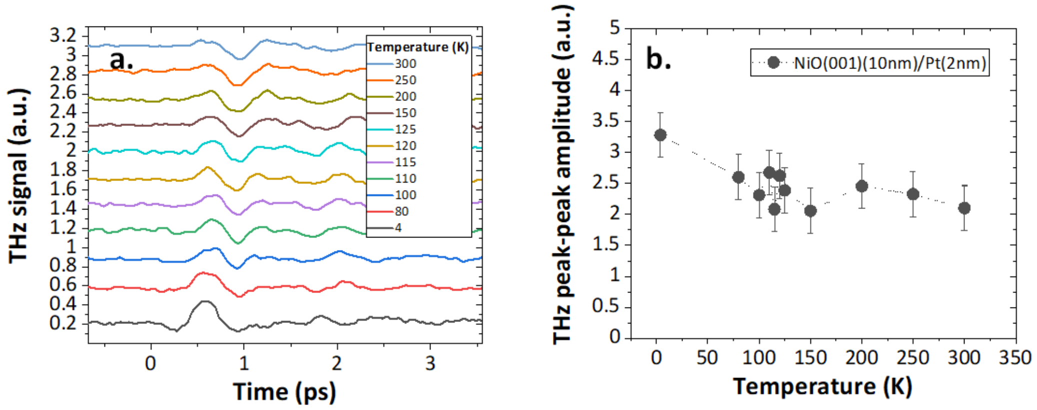

Fig. S14. Temperature dependence of NiO(001)(10nm)/Pt(2nm) THz emission ranging from 4 K to room temperature (300 K). (a) Time-domain THz signal as a function of temperature. Time-domain curves are offset in amplitude for clarity. (b) THz peak-peak amplitude extracted from the THz emission in time-domain as a function of temperature. At low temperatures, the variation allows us to exclude a spin Seebeck effect.

References

[1] F. Schreiber, L. Baldrati, C. Schmitt, R. Ramos, E. Saitoh, R. Lebrun, and M. Kläui, *Concurrent Magneto-Optical Imaging and Magneto-Transport Readout of Electrical Switching of Insulating Antiferromagnetic Thin Films*, Appl. Phys. Lett. **117**, 082401 (2020).

[2] T. Seifert et al., *Efficient Metallic Spintronic Emitters of Ultrabroadband Terahertz Radiation*, Nat. Photonics **10**, 483 (2016).

[3] L. Baldrati et al., *Full Angular Dependence of the Spin Hall and Ordinary Magnetoresistance in Epitaxial Antiferromagnetic NiO(001)/Pt Thin Films*, Phys. Rev. B **98**, 024422 (2018).

[4] C. Schmitt et al., *Identification of Néel Vector Orientation in Antiferromagnetic Domains Switched by Currents in NiO/Pt Thin Films*, Phys. Rev. Appl. **15**, 034047 (2021).

[5] W. L. Roth, *Neutron and Optical Studies of Domains in NiO*, J. Appl. Phys. **31**, 2000 (1960).

[6] I. Sänger, V. V. Pavlov, M. Bayer, and M. Fiebig, *Distribution of Antiferromagnetic Spin and Twin Domains in NiO*, Phys. Rev. B **74**, 144401 (2006).

[7] J. Sinova, S. O. Valenzuela, J. Wunderlich, C. H. Back, and T. Jungwirth, *Spin Hall Effects*, Rev. Mod. Phys. **87**, 1213 (2015).

[8] C. Tzschaschel, K. Otani, R. Iida, T. Shimura, H. Ueda, S. Günther, M. Fiebig, and T. Satoh, *Ultrafast Optical Excitation of Coherent Magnons in Antiferromagnetic NiO*, Phys. Rev. B **95**, 174407 (2017).

[9] H. Sakimura, A. Asami, H. Hayashi, T. Harumoto, Y. Nakamura, J. Shi, and K. Ando, *Intrinsic Spin Decay Length in an Antiferromagnetic Insulator*, Phys. Rev. Res. **1**, 013013 (2019).

[10] H. Qiu et al., *Ultrafast Spin Current Generated from an Antiferromagnet*, Nat. Phys. **17**, 388 (2021).

[11] G. R. Hoogeboom and B. J. van Wees, *Nonlocal Spin Seebeck Effect in the Bulk Easy-Plane Antiferromagnet NiO*, Phys. Rev. B **102**, 214415 (2020).

[12] J. Holanda, D. S. Maior, O. Alves Santos, L. H. Vilela-Leão, J. B. S. Mendes, A. Azevedo, R. L. Rodríguez-Suárez, and S. M. Rezende, *Spin Seebeck Effect in the Antiferromagnet Nickel Oxide at Room Temperature*, Appl. Phys. Lett. **111**, 172405 (2017).

[13] S. M. Rezende, A. Azevedo, and R. L. Rodríguez-Suárez, *Magnon Diffusion Theory for the Spin Seebeck Effect in Ferromagnetic and Antiferromagnetic Insulators*, J. Phys. Appl. Phys. **51**, 174004 (2018).

[14] T. S. Seifert et al., *Femtosecond Formation Dynamics of the Spin Seebeck Effect Revealed by Terahertz Spectroscopy*, Nat. Commun. **9**, 2899 (2018).

[15] T. Higuchi, N. Kanda, H. Tamaru, and M. Kuwata-Gonokami, *Selection Rules for Light-Induced Magnetization of a Crystal with Threefold Symmetry: The Case of Antiferromagnetic NiO*, Phys. Rev. Lett. 4 (2011).

[16] D. Schick, *Udkm1Dsim – a Python Toolbox for Simulating 1D Ultrafast Dynamics in Condensed Matter*, Comput. Phys. Commun. **266**, 108031 (2021).

[17] M. Mattern, A. von Reppert, S. P. Zeuschner, J.-E. Pudell, F. Kühne, D. Diesing, M. Herzog, and M. Bargheer, *Electronic Energy Transport in Nanoscale Au/Fe Hetero-Structures in the Perspective of Ultrafast Lattice Dynamics*, Appl. Phys. Lett. **120**, 092401 (2022).

[18] A. von Reppert, R. M. Sarhan, F. Stete, J. Pudell, N. Del Fatti, A. Crut, J. Koetz, F. Liebig, C. Prietzel, and M. Bargheer, *Watching the Vibration and Cooling of Ultrathin Gold Nanotriangles by Ultrafast X-Ray Diffraction*, J. Phys. Chem. C **120**, 28894 (2016).

[19] R. Shayduk, V. Vonk, B. Arndt, D. Franz, J. Strempfer, S. Francoual, T. F. Keller, T. Spitzbart, and A. Stierle, *Nanosecond Laser Pulse Heating of a Platinum Surface Studied by Pump-Probe X-Ray Diffraction*, Appl. Phys. Lett. **109**, 043107 (2016).

[20] H. Watanabe, *Thermal Constants for Ni, NiO, MgO, MnO and CoO at Low Temperatures*, Thermochim. Acta **218**, 365 (1993).

[21] T. Barron, W. Berg, and J. Morrison, *On the Heat Capacity of Crystalline Magnesium Oxide*, Proc. R. Soc. Lond. Ser. Math. Phys. Sci. **250**, 70 (1959).

[22] M. J. Duggin, *The Thermal Conductivities of Aluminium and Platinum*, J. Phys. Appl. Phys. **3**, L21 (1970).

[23] A. J. Slifka, B. J. Filla, and J. M. Phelps, *Thermal Conductivity of Magnesium Oxide from Absolute, Steady-State Measurements*, J. Res. Natl. Inst. Stand. Technol. **103**, 357 (1998).

[24] R. E. MacFarlane, J. A. Rayne, and C. K. Jones, *Temperature Dependence of Elastic Moduli of Iridium*, Phys. Lett. **20**, 234 (1966).

[25] S. M. Collard and R. B. McLellan, *High-Temperature Elastic Constants of Platinum Single Crystals*, Acta Metall. Mater. **40**, 699 (1992).

[26] N. Uchida and S. Saito, *Elastic Constants and Acoustic Absorption Coefficients in MnO, CoO, and NiO Single Crystals at Room Temperature*, J. Acoust. Soc. Am. **51**, 1602 (1972).

[27] M. A. Durand, *The Temperature Variation of the Elastic Moduli of NaCl, KCl and MgO*, Phys. Rev. **50**, 449 (1936).

[28] R. S. Krishnan, R. Srinivasan, S. Devanarayanan, and B. R. Pamplin, *Thermal Expansion of Crystals: International Series in The Science of The Solid State.* (Elsevier Science, Burlington, 1979).

[29] S. K. Agrawal, J. Narain, and J. Shanker, *Investigation of the Equation of State and the Grüneisen Parameters for Transition Metal Oxides*, Phys. Status Solidi B **123**, 497 (1984).

[30] L. C. Bartel and B. Morosin, *Exchange Striction in NiO*, Phys Rev B **3**, 1039 (1971).

[31] G. K. White and O. L. Anderson, *Grüneisen Parameter of Magnesium Oxide*, J. Appl. Phys. **37**, 430 (1966).

[32] D. Zahn, H. Seiler, Y. W. Windsor, and R. Ernstorfer, *Ultrafast Lattice Dynamics and Electron–Phonon Coupling in Platinum Extracted with a Global Fitting Approach for Time-Resolved Polycrystalline Diffraction Data*, Struct. Dyn. **8**, 064301 (2021).
